# Diagnosing Dysfunctional Voiding Non-Invasively: The SHADE Criteria Approach

**DOI:** 10.5152/tud.2025.25001

**Published:** 2025-07-29

**Authors:** Gautam Shubhankar, Vikas Kumar Panwar, Md Taher Mujahid, Ankur Mittal, Shubham Miglani, Arup Kumar Mandal

**Affiliations:** Department of Urology, All India Institute of Medical Sciences, Uttarakhand, India

**Keywords:** Clinical parameters, dysfunctional voiding, lower urinary tract symptoms, non-invasive diagnosis, urodynamic studies

## Abstract

**Objective:**

Dysfunctional voiding (DV) is an often-underdiagnosed condition primarily affecting younger patients with lower urinary tract symptoms (LUTS). Characterized by a lack of coordination between the detrusor muscle and the external urethral sphincter, DV commonly manifests as urinary frequency, urgency, and incontinence. Despite its significant impact, urodynamic studies (UDS), the gold standard for diagnosis, are frequently inaccessible in remote or under-resourced areas. This study investigates non-invasive clinical parameters to facilitate provisional DV diagnosis.

**Methods:**

A retrospective analysis of 813 patients who underwent UDS for LUTS over 3 years (2021-2024) was conducted. Excluding those with neurological disorders or urethral strictures, 516 patients were evaluated, identifying 67 with DV. Parameters were examined across 2 age groups: under 50 years and 50 years or older, focusing on symptomatology, uroflowmetry, and associated conditions. Statistical analyses, including Chi-square tests and multivariate logistic regression, were employed to identify significant predictors.

**Results:**

Of the 67 patients diagnosed with DV, 64 were under 50 years of age. Statistically significant associations were found between DV and increased diurnal frequency, pre-existing heightened anxiety, obstructive uroflowmetry patterns, constipation, and hypertonic anal sphincter. The proposed non-invasive criteria—SHADE (Staccato/obstructive voiding pattern, Heightened anxiety, Age <50, Diurnal frequency, Exclusion of stricture or neurological disease)—demonstrated over 95% positive predictive value for DV.

**Conclusion:**

Early and accurate diagnosis of DV can be enhanced through non-invasive clinical criteria, particularly in settings where urodynamic testing is limited. Implementing the SHADE criteria can facilitate prompt, targeted management of DV, improving patient outcomes in resource-constrained environments.

**Main Points**

- Early Recognition of Dysfunctional Voiding (DV) through Non-Invasive Parameters: The study demonstrates the utility of the SHADE criteria (Staccato voiding pattern, Hypertonic anal sphincter, Anxiety predating LUTS, Diurnal frequency of micturition, and Exclusion of neurological causes) in identifying DV, reducing reliance on invasive urodynamic studies.
- Association with Anxiety and Improper Toilet Training: Pre-existing heightened anxiety and improper toilet training were found to be significant predictors of DV, highlighting the importance of a detailed psychosocial history in the diagnostic workup.
- Staccato Flow as a Key Indicator: The presence of a staccato uroflowmetry pattern, with or without significant post-void residual, emerged as a consistent finding in over 95% of DV patients, underlining its diagnostic relevance.
- Significance of Constipation and Hypertonic Anal Sphincter: Constipation and hypertonic anal sphincter were significantly associated with DV, indicating the need for multidisciplinary management addressing bowel dysfunction in these patients.
- Reduced Dependency on Urodynamics in Resource-Limited Settings: By employing the SHADE criteria, clinicians in resource-constrained settings can make a provisional diagnosis of DV, improving patient outcomes in areas lacking advanced diagnostic tools.

## Introduction

Dysfunctional voiding (DV) is an underdiagnosed entity usually seen in young patients presenting with lower urinary tract symptoms (LUTS). The incidence of DV is generally 0.5%-2% in patients who are referred to urology centers for non-satisfactory results after mono treatment with alpha-blockers for LUTS.^1^^,^^2^ The International Continence Society (ICS) defines DV as a condition in which there is an intermittent and/or incomplete voiding of urine that is typically due to a lack of coordination between the detrusor muscle of the bladder and the external urethral sphincter. In children, this condition manifests as an inappropriate contraction of the pelvic floor or external urethral sphincter during the voiding phase, leading to difficulties in emptying the bladder and causing a variety of symptoms such as urinary frequency, urgency, incontinence, or urinary tract infections. Urodynamic study testing (UDS) is usually needed in these subsets of patients to reach the final diagnosis. Unfortunately, urodynamic study is an invasive test and is not available at all centers, especially in remote areas where a tertiary care center is not feasible due to multiple factors. Data from the Urology Care Foundation have shown that in sub-Saharan Africa, the availability of specialized urodynamic equipment and trained personnel is extremely low. In Asia, countries like Afghanistan, Bangladesh, and Nepal, which have less developed healthcare infrastructure, particularly in rural areas, also lack this diagnostic capability.^3^ This gives rise to many patients whose diagnoses get missed. As a result, they are usually treated with alpha-blockers by many clinicians, which does not provide them satisfactory improvement in symptoms. This prompted the authors to find clinically non-invasive testing parameters that can be employed to make a provisional diagnosis of DV.^3^

## Material and Methods

The study was approved by Ethics committee of AIIMS Rishikesh (approval number: AIIMS/IEC/25/324, approval date 08/07/2025.) The data of all 813 patients who underwent UDS for evaluation of LUTS at the institute in the urology department over a period of 3 years (2021-2024) was retrospectively analyzed. Traditional knowledge reiterates that DV is a diagnosis made on UDS. So, in the same context, DV, as per the ICS, on UDS was defined as an increase in the EMG (Electromyography) activity during the voiding phase without any concomitant increase in abdominal pressure (Pabd) in patients without any known neurological cause of LUTS.^4^

**Figure 1.**
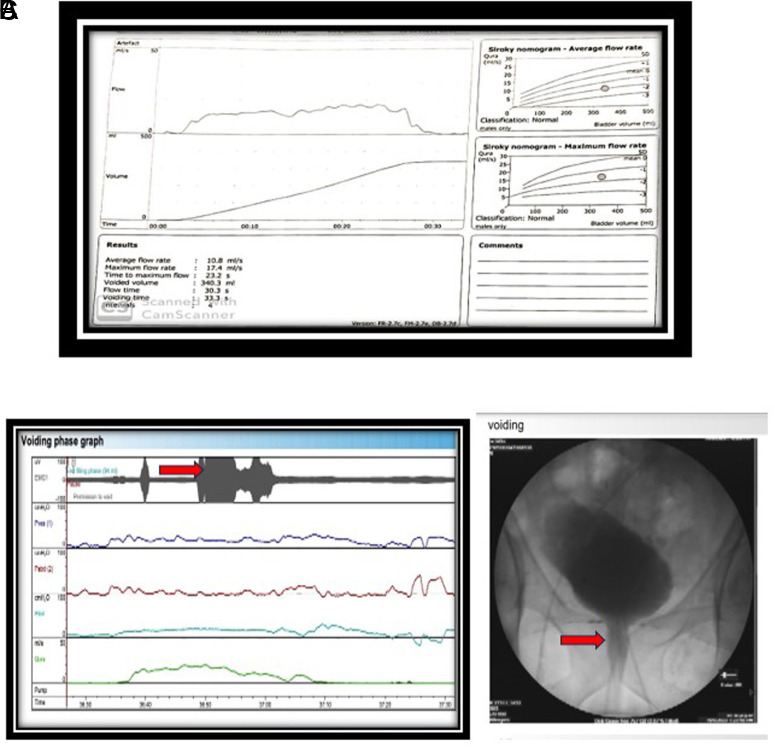
Showing typical uroflowmetry and video urodynamic study findings in dysfunctional voiding.

We, therefore, excluded 297 patients who had pre-existing known neurological diseases causing LUTS, post-spinal trauma or spinal surgery cases, and patients with a history of urethral stricture or multiple urethral interventions done earlier. Out of the remaining 516 patients, it was finally found that a total of 67 patients were diagnosed with DV. In this study, the diagnosis of DV was made using a video urodynamic study (VUDS). Prior to initiating the urodynamic study, the apparatus was well calibrated, and cough test was essentially done to verify the transmission of the pressures. All those patients without any known neurological cause of LUTS who had an increase in the EMG activity (in the absence of any abdominal straining or a concomitant rise of Pabd) with high pressure [Detrusor pressure (Pdet) >60 mm Hg] and low flow [Maximum flow rate (Qmax) < 15 mL/sec or Bladder outlet obstruction (BOO) index > 25] voiding pattern during the voiding phase of VUDS, with the typical spinning top deformity at the bladder outlet during the micturating cystourethrogram phase were diagnosed as having DV (Figure 1). For comparison, patients with bladder neck dysfunction as part of the non-dysfunctional voiding (NDV) group were also considered. So, there was an incidence of DV in ~11% of the total patients, which was similar to that in the literature. On further reviewing the demographic features of these patients, it was found that 64 (>95%) of them were <50 years of age. Moreover, the 3 patients in the age group >50 years had the onset of LUTS before 50. Also, among the patients >50 years of age included in the study, 3 were diagnosed with DV and 382 had diagnoses other than DV (Table 1). This predominance of DV in the <50 years age group led to reckon if there occurs any association of DV with age, to begin with.

For statistical analysis, SPSS software version 28 was used. Upon using the Chi-square distribution table with 1 degree of freedom, the *P*-value for the calculated *χ*^2^ = 199.7 came out to be <.05, indicating a significant association of DV with <50 years of age.

This statistically significant correlation of DV with <50 years of age led to segregation of the patients into <50 years and >50 years age groups and carry on with the further analysis of parameters among the 131 patients of the <50 years age group. Among the <50 years age group, 3 patients were <16 years and the remaining 128 patients were of the 16-50 years age group. So, ultimately, these 131 patients were divided into 2 groups (based on UDS diagnoses):

Group I: Dysfunctional voiders (64 patients) and

Group II: Non-dysfunctional voiders (67 patients) (having UDS diagnoses other than DV) (Figure 1).

**Figure 2.**
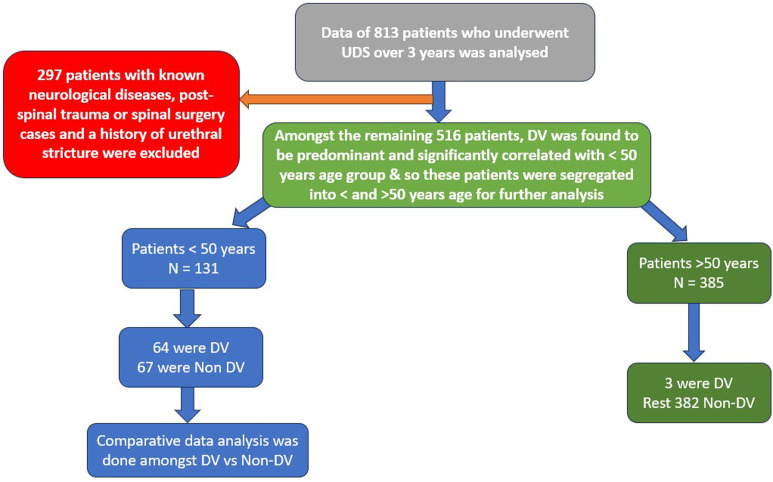
Showing the study algorithm.

The non-dysfunctional voiders comprised 24 (21%) patients diagnosed as underactive detrusor, 7 (6%) as overactive bladder, and 19 (17%) as BOO secondary to other etiologies, which during cystoscopy were found to be high bladder neck 8 (7%) and primary bladder neck obstruction (PBNO) 11 (10%) (Figure 2).

*Z* test and Student *t*-tests were used for testing the significance of the incidence of different parameters in the 2 groups. Univariate and multivariate analysis was done following that between the significant parameters, and the results were concluded.

## Results

**Figure 3.**
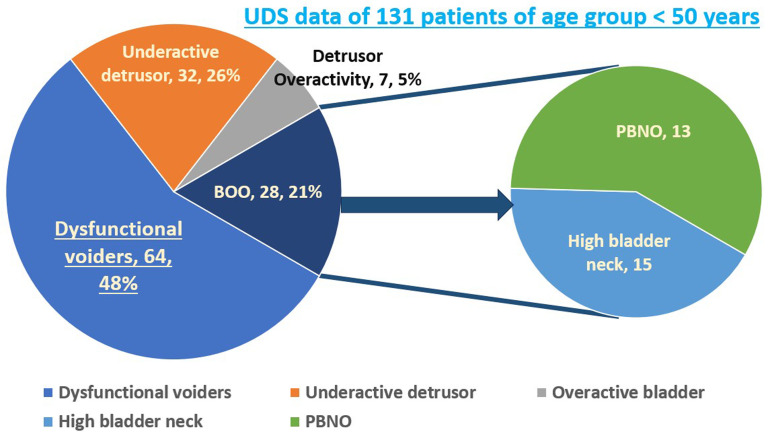
Showing the urodynamic studies diagnosis of the 114 patients.

A comprehensive study regarding the detailed history and relevant findings on clinical examination, IPSS score and quality of life, uroflowmetry with post-voided residual volume (PVR) values, and urodynamic study parameters was done and analyzed. Group I (Dysfunctional Voiders) and Group II (Non-Dysfunctional Voiders) were analyzed to compare clinical features. Non-dysfunctional voiding patients included those with underactive detrusor, overactive bladder, or BOO due to high bladder neck or PBNO (Figure 3). Detrusor overactivity refers to involuntary contractions during the filling phase, confirmed by UDS, while overactive bladder is a clinical diagnosis based on urgency symptoms.

Data of 131 patients, out of the 813 patients who underwent urodynamic testing, who met the inclusion criteria, were analysed under the following headings:

Baseline Characteristics: Among the dysfunctional voiders (DV) group, 80% of the patients were males and the remaining 20% were females, whereas in the non-dysfunctional voiders group, 78% were males and the remaining 22% were females. This difference was statistically non-significant. The age group intervals of the patients in the DV group were distributed as follows: 5 patients in the 10-20 years group, 10 patients in the 20-30 years group, 25 patients in the 30-40 years group, and 24 patients in the 40-50 years group. The mean ages of the patients in the DV and NDV groups were 34.7 years and 35.8 years, respectively. This difference was also not statistically significant.

Symptomatology and Uroflowmetry: It was found that frequency (diurnal) was significantly higher in the DV group, whereas nocturia was significantly higher in the NDV group. Nocturia, even though present in a few dysfunctional voiders, was not bothersome, unlike the patients with NDV, particularly overactive bladder, who had bothersome nocturia in almost all the patients. The rest of the parameters were statistically non-significant (Table 2).

A uroflowmetry pattern suggestive of a staccato (compressive) pattern was seen in 62 (>95%) of the patients in the DV group and 34 (~50%) patients in the non-DV group, and this was found to be statistically significant (*P*-value <.05). Such a pattern of flow included all those patients wherein the maximum flow rate (Qmax) was achieved after 10 seconds of giving a voiding command or Qmax attained after the initial 1/3rd of the voiding curve. Also, the average flow rates (Qavg) of such curves were almost half of the Qmax.

Among the dysfunctional voiders, it was seen that 91% of them had pre-existing heightened anxiety, significantly impacting their quality of life, before the onset of LUTS. Heightened anxiety was defined as a score >4 on the Generalized Anxiety Disorder 7 (GAD-7) scoring system, which is a patient-reported questionnaire. In the rest of the patients with other UDS diagnoses, this anxiety element, even though present, had its onset after the onset of LUTS. Inquiring about this anxiety element is crucial to figure out in history taking and is often missed or ignored. However, making the patient comfortable with the surroundings, followed by a confidently humble tone in history taking, can help identify the underlying anxiety component or improper toilet training given by their parents. Pre-existing heightened anxiety was seen in only in 5% of patients with non-dysfunctional voiders, and again this difference was statistically significant (*P*-value < .0001).

Constipation was seen in ~73% and hypertonic anal sphincter in ~35% of the dysfunctional voiders. Constipation was defined using the Rome IV criteria as having at least 2 or more of the following in over 25% of defecations: straining, hard/lumpy stools, incomplete evacuation, anorectal obstruction, or the need for manual assistance. Patients must have fewer than 3 spontaneous bowel movements per week, with symptoms lasting at least 3 months and starting at least 6 months prior. Hypertonic anal sphincter was defined as the anal tone that does not admit even 1 finger for digital rectal examination without significant pain. Among the non-dysfunctional voiders, constipation and hypertonic anal sphincter were seen in 42% and 6% respectively. This difference in the proportion of patients with constipation and hypertonic anal sphincter in the 2 groups was statistically significant. Furthermore, a staccato pattern on uroflowmetry with or without significant PVR was seen in 95% of the dysfunctional voiders and 64% of the non-dysfunctional voiders respectively, and this difference too was statistically significant (Table 1).

To conclude, it was found that the statistically significant parameters upon the comparison of the 2 groups and in support of DV were: increased diurnal frequency of micturition, history of heightened anxiety significantly affecting the quality of life with its onset before LUTS, obstructive voiding pattern on uroflowmetry with or without significant PVR with the exclusion of urethral stricture disease, and a known neurological cause of LUTS. A univariate analysis of these 5 parameters revealed an odds ratio of > 1 and a *P*-value of < .05. Further performing a multivariate logistic regression showed that these 5 variables together significantly predict the diagnosis of DV (with ORs > 1 and *P*-values < .05).

## Discussion

The ICS defines dysfunctional voiding (DV) as a functional voiding disorder where individuals habitually contract the urethral sphincter during urination without a neurological cause, typically presenting with a staccato uroflowmetry pattern. This dysfunction leads to symptoms such as urinary incontinence, urgency, frequency, and recurrent urinary tract infections (UTIs). While DV is more common in females, 5%-10% of children with voiding dysfunction are boys, predominantly aged 5-15 years, with a peak between 5 and 10 years. The incidence decreases with age but may persist into adulthood. This study included patients under 50 years old, covering the most affected age group. Since DV involves bladder-sphincter discoordination, bladder neck dysfunction should be considered in young males with similar symptoms.^5^^,^^6^

Studies by Gatti and Perez-Brayfield^5^ and Koff and Wagner^8^ showed that symptoms of DV in young males are particularly impactful, frequently manifesting as daytime urinary incontinence, nocturnal enuresis (bedwetting), and a persistent sense of urinary urgency and frequency. Some boys also experience recurrent UTIs due to incomplete bladder emptying, further complicating their health. These symptoms can profoundly affect their quality of life, leading to social embarrassment, heightened anxiety, and various behavioral issues that can extend into their daily activities and interactions. Several factors contribute to the development of DV in young males. Behavioral aspects, such as inadequate toilet training, deliberate urine withholding, and psychosocial stressors, play a significant role. A similar finding was noted in this study wherein more than 90% of the young patients who had an increased daytime frequency of voiding were finally diagnosed as dysfunctional voiders. Also, this study revealed that more than 90% of patients who were diagnosed as dysfunctional voiders had pre-existing heightened anxiety issues or stress impacting their quality of life.^6^^,^^7^

Constipation frequently accompanies DV, significantly complicating the clinical picture and management of affected patients. In pediatric populations, the prevalence of constipation in children with DV is notably high, with studies indicating that up to 50% of these children also suffer from constipation. Constipation can exacerbate voiding dysfunction by increasing abdominal pressure, adversely impacting bladder function, and leading to incomplete bladder emptying. Studies, such as those by Koff and Wagner^8^, reveal that addressing constipation can lead to significant improvements in DV symptoms, highlighting the importance of a comprehensive approach to managing these intertwined conditions. Likewise, in this study, it was found that ~60% of the patients diagnosed with dysfunctional voiders had constipation.^8^

A hypertonic anal sphincter, characterized by increased muscle tone in the anal sphincter, is a common and significant finding in individuals with DV. This condition frequently leads to chronic constipation, exacerbating the symptoms of DV and complicating its clinical management. In pediatric populations, the prevalence of a hypertonic anal sphincter among children with DV is striking, with research indicating that up to 30%-50% of these young patients are affected. The significant overlap between constipation and a hypertonic anal sphincter in children with DV further complicates their condition, as the increased muscle tone hampers stool passage and intensifies urinary symptoms due to the additional pressure on the bladder and surrounding structures. These symptoms not only cause discomfort but also aggravate DV by increasing intra-abdominal pressure and impairing normal voiding mechanisms. Clinical studies, notably those by Loening-Baucke and colleagues, have meticulously documented the prevalence and impact of a hypertonic anal sphincter in children with DV. Their findings underscore the critical importance of addressing both urinary and bowel symptoms comprehensively to achieve optimal management and improve the quality of life for these young patients. Similarly, in this study, ~50% of the patients who had DV had increased anal sphincter tone.^9^

An obstructive/staccato outflow pattern on uroflowmetry is a prominent finding in patients with DV. This distinct pattern reveals uncoordinated contractions between the bladder and sphincter muscles, leading to a disrupted urinary stream. In pediatric populations, the staccato pattern serves as a crucial diagnostic indicator, with studies estimating its presence in up to 40%-60% of children with DV. Its clinical relevance is profound, aiding clinicians in confirming DV diagnoses, gauging severity, and devising targeted treatment plans to enhance bladder-sphincter coordination. Children displaying a staccato pattern often endure symptoms such as urinary urgency, frequency, incontinence, and incomplete bladder emptying, which can result in recurrent UTIs and significantly diminish their quality of life. Pioneering research, including studies by Nevéus et al,^10^ underscores the prevalence and implications of the staccato pattern, highlighting the indispensable role of uroflowmetry in the effective diagnosis and management of DV. This study, similarly, revealed that more than 90% of the patients diagnosed as dysfunctional voiders showed a staccato pattern on uroflowmetry with or without significant PVR.

Diagnosis of DV is made on the UDS. However, a UDS is an invasive procedure and is not available everywhere, especially in remote areas. Also, learning and interpretation of UDS with the demonstration of increased EMG activity during voiding without a rise in abdominal pressure are tricky for many clinicians.^11^ Many of these patients are hesitant to void during the study, making the diagnosis difficult. Keeping all these practical issues in mind, a multivariate analysis of the different variables related to DV was conducted.

It was derived that those patients who fulfilled all the following pentad of criteria:

S – Staccato voiding pattern on UFM (uroflowmetry) suggesting outflow obstruction

H – Heightened anxiety, having onset before LUTS, impacting quality of life

A – Age <50 years

D – Diurnal frequency of micturition without bothersome nocturia if present.

E – Exclusion of patients with urethral stricture disease or pre-existing neurological disease,

had almost a 95% chance of having an underlying DV. This was validated by calculating the positive predictive value for diagnosing DV (using the above parameters) with a Bayesian statistical model. In this model, a sensitivity of 90% and a specificity of 95% were applied, with a prevalence of 40%. Using these parameters, the PPV (positive predictive value) was found to be 95%.

Employing this in clinical practice, therefore, would help clinicians to provisionally diagnose DV. Although constipation and hypertonic anal sphincter were significantly more prevalent in DV patients (73% and 35%, respectively), they were excluded from SHADE due to their lack of specificity and difficulty in routine assessment. Instead, these should be addressed during treatment. Such patients should be started with the DV-targeted treatment right from the beginning (especially where urodynamic testing is not easily available or due to the long waiting time for UDS at high volume centers) which would include – lifestyle modifications like meditation and yoga, avoidance of caffeinated beverages, avoiding fluid intake after 8 pm; pelvic floor relaxation exercises with biofeedback sessions; alpha-blockers; sitz baths for patients with hypertonic anal sphincter; avoidance of constipation; and central muscle relaxants like tablet baclofen if needed. After starting this DV-guided treatment, a follow-up should be done after 3-4 weeks, which includes, IPSS symptom score reassessment, a repeat uroflowmetry with PVR, regular biofeedback sessions, and ultrasound for bilateral upper tract assessment for any hydroureteronephrosis.

The study has a few limitations. It was a single-center study and the sample size was small. Following this approach will aid in the diagnosis to start some treatment empirically on the lines of DV in a majority of the patients, especially at centers where UDS facility is not available, but a few patients might not turn out to be DV. The investigation of choice still remains UDS, if done aptly, for diagnosing DV.

The study demonstrates that the SHADE criteria, comprising staccato voiding pattern, heightened anxiety, age under 50 years, diurnal frequency without bothersome nocturia, and exclusion of urethral stricture or pre-existing neurological disease, offers a non-invasive framework for diagnosing detrusor underactivity (DV). These criteria provide a predictive power of over 95%, highlighting their potential as an effective tool for early diagnosis. The application of the SHADE criteria can enable earlier treatment of DV, particularly in settings where urodynamic tests are less accessible or where there are long waiting times. However, these findings need further validation in broader, diverse populations before widespread clinical implementation.

## Figures and Tables

**Table 1.** Showing the Incidence of Dysfunctional Voiding in <50 and >50 Years of Age Groups Devoid of the Exclusion Criteria

Age Group	Dysfunctional voiding Present	Dysfunctional voiding Absent	Total
<50 years	64	67	131
>50 years	3	382	385
Total	67	449	516

**Table 2.** Showing Comparison of Parameters Among the Dysfunctional Voiding and Non-Dysfunctional Voiding Groups <50 Years of Age

	Dysfunctional Voiders (<50 years age) (n = 64)	Non-Dysfunctional Voiders (<50 years age) (n = 67)	*P*
Frequency	59 (92.1%)	23 (35%)	<.001
Urgency	34 (53.1%)	25 (37%)	.31
Nocturia	19 (29.6%)	34 (50%)	<.0001
Poor flow	28 (43.7%)	32 (48%)	.53
Intermittency	31 (48.4%)	35 (51%)	.08
Incomplete evacuation	48 (75%)	37 (55%)	.9
Straining	46 (71.8%)	36 (52%)	.9
IPSS (Mean%)	17.7	18.68	.28
Quality of life	2.68	2.98	.08
Q max	12.72	11.88	.39

## Data Availability

Data have been made available from the retrospective analysis of the young patients who have undergone urodynamic study over a period of 3 years after concurrence with the institute.
